# Time trends in anxiety disorders incidence across the BRICS: an age-period-cohort analysis for the GBD 2021

**DOI:** 10.3389/fpubh.2024.1467385

**Published:** 2024-10-07

**Authors:** Dan Liu, Murong Luo, Yan Huang, Yingfang Tan, Fangqun Cheng, Yuhang Wu

**Affiliations:** ^1^Xiangtan Central Hospital, Xiangtan, China; ^2^Department of Epidemiology and Health Statistics, Xiangya School of Public Health, Central South University, Changsha, China

**Keywords:** anxiety disorders, incidence, age-period-cohort model, BRICS, time trends

## Abstract

**Background:**

Anxiety disorders are a significant global mental health concern, contributing to substantial disability-adjusted life years (DALYs) and imposing considerable social and economic burdens. Understanding the epidemiology of anxiety disorders within the BRICS nations (Brazil, Russian Federation, India, China, and South Africa) is essential due to their unique socio-economic landscapes and ongoing transformations.

**Methods:**

This study utilized data from the Global Burden of Disease (GBD) 2021 database to evaluate anxiety disorder incidence trends in BRICS countries from 1992 to 2021. The Age-Period-Cohort (APC) model with an intrinsic estimator (IE) algorithm was employed to disentangle the effects of age, period, and cohort on incidence rates. Data were categorized into 5-year age groups, and 95% uncertainty intervals (UIs) were calculated to account for data variability.

**Results:**

From 1992 to 2021, the global number of anxiety disorders cases increased by 73.44%, with age-standardized incidence rates rising by 21.17%. Among BRICS nations, India experienced the largest increase in cases (113.30%), while China had the smallest increase (2.79%). Globally, young (15–49 years) and oldest (80–94 years) age groups showed predominantly positive local drift values, indicating rising incidence rates. Brazil and India mirrored this trend, while China and South Africa mostly exhibited negative local drift values. Russia Federation had mixed trends with younger groups showing negative and older groups positive local drift values. The incidence of anxiety disorders exhibited an “M-shaped” age pattern with peaks at 10–14 and 35–39 years. Period effects were stable globally but varied in BRICS countries, with Brazil showing a decline and India an increase. Cohort effects were stable globally but showed increasing trends in Brazil and India post-1955–1959 cohort.

**Conclusion:**

This study highlights a significant increase in anxiety disorders incidence globally and within BRICS nations over the past three decades, with marked variations across countries. The distinct trends observed in age, period, and cohort effects call for age-specific and gender-sensitive mental health policies. Continuous monitoring, research, and tailored public health strategies are essential to address the rising burden of anxiety disorders and improve mental health outcomes in these rapidly evolving regions.

## Introduction

Anxiety disorders are characterized by experiences of intense fear and distress, typically in combination with other physiological symptoms, constitute a significant mental health concern globally. These disorders are among the leading causes of disability-adjusted life years (DALYs) and affect millions (1, 2), imposing substantial social and economic burdens (3, 4). The epidemiology of anxiety disorders is influenced by a myriad of factors including genetic predispositions, environmental exposures, and socio-cultural contexts, leading to varied incidence rates across different regions (2, 5).

Focusing on the BRICS nations (Brazil, Russian Federation, India, China, and South Africa) is particularly pertinent due to their unique socio-economic landscapes, immense population sizes, and rapid transformations over recent decades (6). These countries collectively represent a significant portion of the world’s population and are undergoing socio-economic shifts that could impact mental health trajectories differently compared to more developed regions (7). Studying anxiety disorders within these nations is crucial for understanding how factors such as urbanization, healthcare infrastructure, and socio-economic disparities uniquely influence mental health in large, diverse populations with differing cultural backgrounds.

The Global Burden of Disease (GBD) 2021 study offers an updated and comprehensive dataset for assessing the burden of anxiety disorders, providing critical insights into temporal trends and geographical variations. This dataset includes extensive data on disease incidence, prevalence, mortality, and attributable risk factors, collected from a wide range of sources worldwide (8–11). GBD 2021’s robust methodology ensures rigorous estimates and comparability across different regions and time periods, making it a valuable resource for examining the epidemiology of anxiety disorders. Traditional epidemiological analyses based on earlier GBD data, such as those from 2019 (2), have contributed valuable global perspectives but often fall short in capturing national-level nuances that are essential for effective policy making. The Age-Period-Cohort (APC) model, by contrast, offers a robust framework for dissecting the complex interplay of aging (Age effect), temporal changes (Period effect), and generational influences (Cohort effect) on disease incidence. This modeling approach is particularly well-suited for revealing temporal patterns and underlying factors driving disease trends in specific populations (12). Despite the utility of the APC model, previous applications, including a study covering the period from 1990 to 2019 (5), provided a global overview of anxiety disorder trends but did not sufficiently address the significant heterogeneity and local differences within individual countries, especially within the BRICS nations. Such granularity is essential for understanding country-specific epidemiological dynamics and tailoring public health interventions.

This study aims to fill these gaps by utilizing the latest GBD 2021 data to conduct an APC analysis focused explicitly on the BRICS countries. By leveraging this data, we seek to provide a more nuanced and detailed understanding of the temporal and contextual factors affecting anxiety disorder incidence at the national level within these countries. The significance of this research is multifold: it enhances the precision of epidemiological insights by addressing country-specific details, thereby informing more tailored and effective public health strategies. Ultimately, the outcomes of this study will help policymakers and healthcare professionals develop targeted interventions and allocate resources more efficiently, thereby improving mental health outcomes for populations within the BRICS nations.

## Method

### Data sources

This study utilized the GBD 2021 public dataset, accessible via the Global Health Data Exchange GBD Results Tool.^1^ The GBD 2021 provides comprehensive insights into the burden of 371 different health conditions across 204 countries and territories, encompassing a broad spectrum of health data related to disease incidence, prevalence, mortality, and risk factors (8–11). The database includes several enhancements, such as the integration of 19,189 additional sources of disability-adjusted life years (DALY) data, the inclusion of 12 new health conditions, and various methodological improvements (11). Additionally, it accounts for the effects of the COVID-19 pandemic on global disease burden estimates.

In the GBD 2021, all cases of anxiety disorders reaching diagnostic threshold defined by the Diagnostic and Statistical Manual of Mental Disorders (DSM) or the World Health Organization (WHO) International Classification of Diseases (ICD) were captured (13, 14). The specific anxiety disorders included were panic disorder, agoraphobia, specific phobia, social phobia, obsessive-compulsive disorder (OCD), post-traumatic stress disorder (PTSD), generalized anxiety disorder (GAD) including overanxious disorder in childhood, separation anxiety disorder (SAD), and anxiety disorder “not otherwise specified” (NOS). The diagnostic classification was ascertained through specific codes in these publications: for DSM-IV-TR: 300.0–300.3, 208.3, 309.21, 309.81; for ICD-10: F40–42, F43.0, F43.1, F93.0–93.2, F93.8. Anxiety disorders attributable to a general medical condition or directly caused by substances were not included in this study. We recognized various editions of the diagnostic manuals including DSM-III, DSM-III-R, DSM-IV, DSM-IV-TR, DSM-5, and DSM-5-TR, as well as ICD-9, ICD-10, and ICD-11 for inclusivity.

We obtained data on the incidence, all-age incidence rates, and age-standardized incidence rates of anxiety disorders globally and specifically within BRICS nations, spanning different age groups over a period from 1992 to 2021. To account for data variability, 95% uncertainty intervals (UIs) were calculated by repeating the data sampling 1,000 times, using the 2.5th and 97.5th percentiles to define the interval boundaries (11). Detailed methodologies and the modeling approach for GBD 2021 were documented in separate publications (10, 11). The dataset used was anonymized and freely accessible to the public; the Institutional Review Board at the University of Washington approved the waiver of informed consent.

### Statistical analysis

#### Age-period-cohort modeling analysis

We employed the APC analytical framework to dissect the dataset, with age, period, and cohort serving as the primary independent variables. The APC model is particularly adept at evaluating the complex interactions between these variables and identifying how they collectively influence the incidence of anxiety disorders. This approach surpasses traditional epidemiological models by providing a multidimensional perspective on disease progression and susceptibility. Furthermore, the intrinsic estimator (IE) method associated with the APC model was applied to addresses the issue of parameter indeterminacy inherent in the APC model’s age, period, and cohort effects. More methodological information is available in the previous literature (15). The key output metrics from the APC model with IE algorithm include net drift, local drift, the longitudinal age curve, and period and cohort relative risks (RR). Net drift captures the overall log-linear trend of the incidence rate across the entire population by both period and cohort. Local drift outlines the trend for each specific age bracket. The longitudinal age curve depicts the expected age-specific rates for a reference cohort, adjusted for period effects. Period RR and cohort RR measure relative risks across different periods and cohorts, respectively, adjusting for both age and the other comparative (period or cohort) (16).

#### Data arrangement

Uniform formatting of age and period data was essential for the structure of the APC model. Therefore, the incidence and population data for anxiety disorders were stratified into predefined categories. Age was segmented into continuous 5-year intervals (0–4, 5–9, 10–14, …, 90–94). The analysis focused on incidence and population data collected at six distinct time points (1994, 1999, 2004, 2009, 2014, and 2019), rather than averaging five-year periods, to better represent each time span. Birth cohorts were determined using the formula: cohort = period – age. This yielded cohorts ranging from 1900–1904 (median year 1902) to 2000–2005 (median year 2003). APC analyses were conducted using the National Cancer Institute’s age-period-cohort web-based tool, with subsequent data visualization and statistical analysis performed in R (version 4.2.3). Statistical inference for parameter significance was carried out using the Wald χ^2^ test, with all tests being two-tailed.

## Results

### Incidence of anxiety disorders trends from 1992 to 2021

Table 1 presents the population, total incidence, all-age incidence rate, age-standardized incidence rate, and net drift of incidence for the world and BRICS countries. Over the past three decades, the number of anxiety disorders cases increased from 31,087 thousand (95% UI 25760 to 38,402) in 1992 to 53,917 thousand (95% UI 44991 to 65,979) in 2021, corresponding to a 73.44% increase. The age-standardized incidence rate increased from 559.73 (95% UI 465.56 to 682.02) per 100,000 population in 1992 to 678.25 (95% UI 565.15 to 832.44) per 100,000 population in 2021, indicating a 21.17% increase. The APC model estimated a net drift of 0.01% (95% confidence interval [CI] -0.04 to 0.07) in the anxiety disorders incidence rate from 1992 to 2021 globally (Table 1).

**Table 1:** Trends in anxiety disorders incidence across BRICS, 1992–2021.

	Global		Brazil		Russia Federation		India		China		South Africa	
	1992	2021	1992	2021	1992	2021	1992	2021	1992	2021	1992	2021
**Population**												
Number, *n* × 1,000,000	5,497 (5,379, 5,624)	7,891 (7,668, 8,131)	153 (142, 165)	220 (188, 251)	152 (138, 166)	145 (125, 164)	885 (819, 951)	1,415 (1,240, 1,602)	1,206 (1,111, 1,302)	1,423 (1,319, 1,530)	39 (35, 42)	57 (50, 64)
Percentage of global, %	100.0	100.0	2.8	2.8	2.8	1.8	16.1	17.9	21.9	18.0	0.7	0.7
**Incidences**												
Number, *n* × 1,000	31,087 (25,760, 38,402)	53,917 (44,991, 65,979)	1,359 (1,119, 1,646)	2,705 (2,218, 3,335)	793 (664, 949)	978 (806, 1,160)	4,044 (3,338, 4,968)	8,626 (7,051, 10,468)	6,513 (5,354, 7,930)	7,734 (6,459, 9,304)	225 (184, 276)	449 (365, 556)
Percentage of global, %	100.0	100.0	4.4	5.0	2.6	1.8	13.0	16.0	21.0	14.3	0.7	0.8
Percent change of number 1982–2021, %	73.44		99.04		23.33		113.30		18.75		99.56	
**All-age incidence rate**												
Rate per 100,000	565.50 (468.61, 698.58)	683.25 (570.13, 836.09)	887.02 (730.02, 1073.51)	1227.56 (1006.62, 1513.56)	522.48 (437.70, 625.10)	674.93 (556.25, 800.86)	456.80 (377.06, 561.16)	609.80 (498.48, 740.02)	539.90 (443.87, 657.39)	543.57 (453.98, 653.92)	582.55 (476.67, 713.06)	789.68 (642.45, 978.20)
Percent change of rate 1992–2021, %	20.82		38.39		29.18		33.49		0.68		35.56	
**Age-standardized incidence rate**												
Rate per 100,000	559.73 (465.56, 682.02)	678.25 (565.15, 832.44)	857.11 (708.70, 1034.56)	1209.77 (993.15, 1503.98)	513.79 (430.14, 620.82)	703.82 (582.55, 842.15)	474.34 (395.82, 572.22)	579.94 (480.12, 702.24)	531.67 (447.77, 632.97)	546.51 (454.19, 662.14)	573.15 (475.30, 693.55)	758.65 (619.89, 932.49)
Percent change of rate 1992–2021, %	21.17		41.15		36.99		22.26		2.79		32.31	
**APC model estimates**												
Net drift of incidence rate*, % per year	0.01 (−0.04, 0.07)		0.21 (−0.17, 0.60)		0.02 (0.01, 0.04)		0.10 (−0.28, 0.48)		−0.35 (−0.61, −0.10)		−0.02 (−0.15, 0.12)	

All-age incidence = crude incidence rate. Age-standardized incidence rate is computed by direct standardization with global standard population in GBD 2021. Net drifts are estimates derived from the age-period-cohort model and denotes overall annual percentage change in incidence, which captures the contribution of the effects from calendar time and successive birth cohorts.

^*^Parentheses for all GBD health estimate indicate 95% uncertainty intervals; parentheses for net drift indicate 95% confidence intervals; APC, age-period-cohort.

The incidence of anxiety disorders cases has significantly increased in all BRICS countries. Notably, India has demonstrated the most substantial increase, at 113.30%. In 2021, the all-age incidence rate and age-standardized incidence rate for anxiety disorders varied from 543.57 (95% UI 453.98 to 653.92) and 546.51 (95% UI 454.19 to 662.14) per 100,000 population in China to 1227.56 (95% UI 1006.62 to 1513.56) and 1209.77 (95% UI 993.15 to 1503.98) per 100,000 population in Brazil, respectively. All the BRICS countries have exhibited an upward trend from 1992 to 2021. Among these nations, Brazil has experienced the most significant increase, with a rate of 41.15%, while China has shown the least significant increase, at 2.79%. According to the APC model estimates, the annual net drift in the anxiety disorders incidence rate ranged from −0.35% (95% UI-0.61 to −0.10) for China to 0.21% (95% UI -0.17 to 0.60) for Brazil within the BRICS countries (Table 1).

### Time trends in anxiety disorders incidence across different age groups

**Figure 1:**
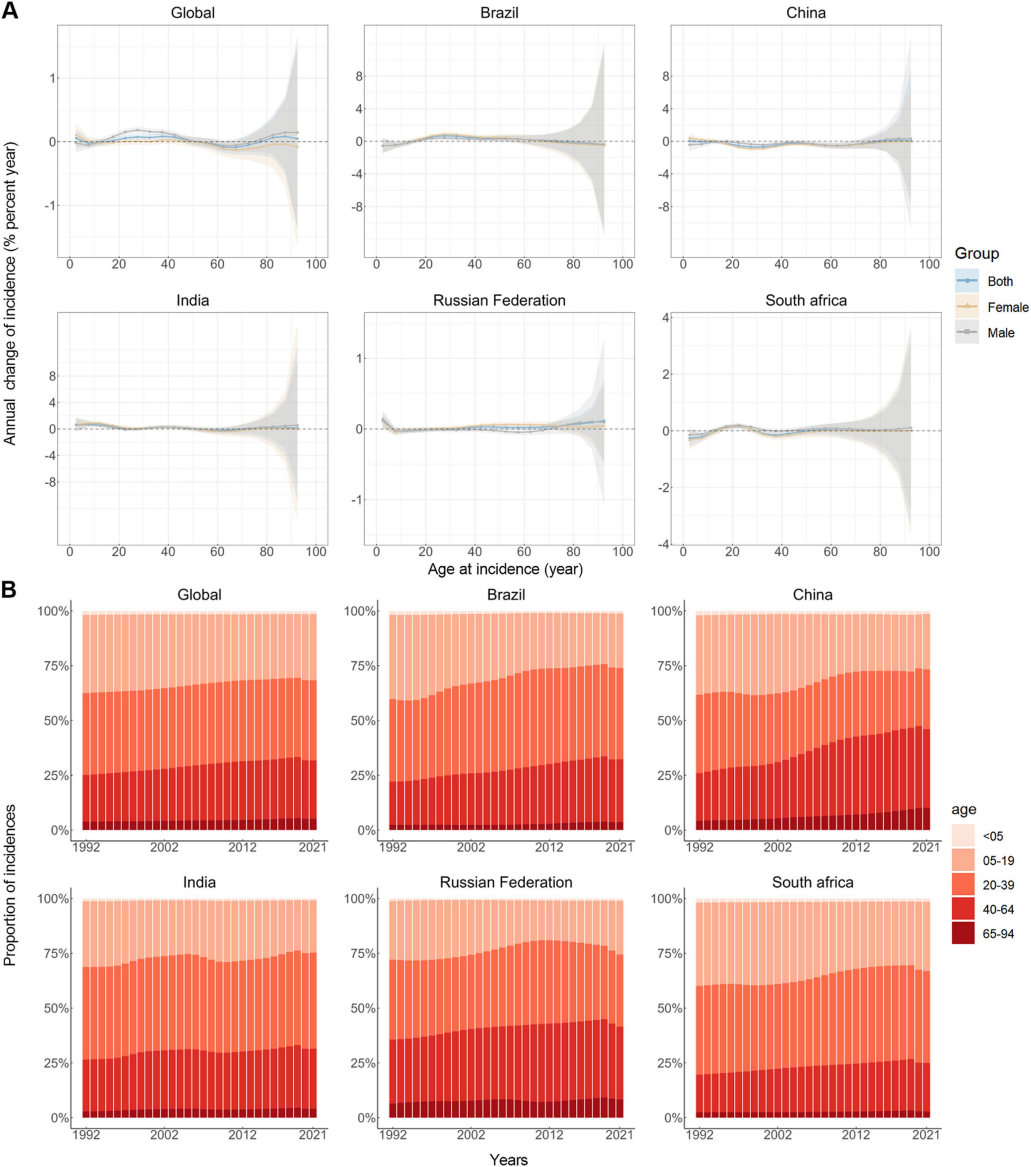
Local drifts of incidence rate and age distribution of incidences in global and BRICS, 1992–2021. **(A)** Local drifts of anxiety disorders incidence rate (estimates from age-period-cohort models) for age groups (0–4, 5–9, 10–14, …, 90–94 years), 1992–2021. The dots and shaded areas indicate the annual percentage change of incidence rate (% per year) and the corresponding 95% CIs. **(B)** Temporal change in the relative proportion of anxiety disorders incidences across age groups, 1992–2021.

Figure 1A depicts the slight annual percentage change in the incidence rates of anxiety disorders for each 5-year age group ranging from 0 to 94 years. Specifically speaking, the young and the oldest age groups (15–49 and 80–94 years) demonstrated predominantly positive local drift values, indicating a rise in the incidence rate of anxiety disorders for these groups, globally. A parallel trend was observed in Brazil and India. China and South Africa exhibited negative local drift values for most age groups, suggesting a decline in the incidence rates of anxiety disorders. In Russia Federation, negative local drift values were observed for lower age groups (under 30) and positive local drift values for higher age groups (30 and over). It should also be noted that males have more age groups associated with positive net drift values than females. Figure 1B illustrates the temporal trends in the number of anxiety disorders cases by age group. Overall, the majority of global anxiety disorders cases were recorded among the young females (5–64 years), and comparable distributions were observed across all BRICS countries. Concurrently, the age distribution of anxiety disorders cases is relatively stable globally and in BRICS nations from 1992–2021, but there was an emerging transition of incidences from the young population (5–19 years) to the middle-aged and old population (20–94 years).

### Age, period and cohort effects on anxiety disorders incidence

**Figure 2:**
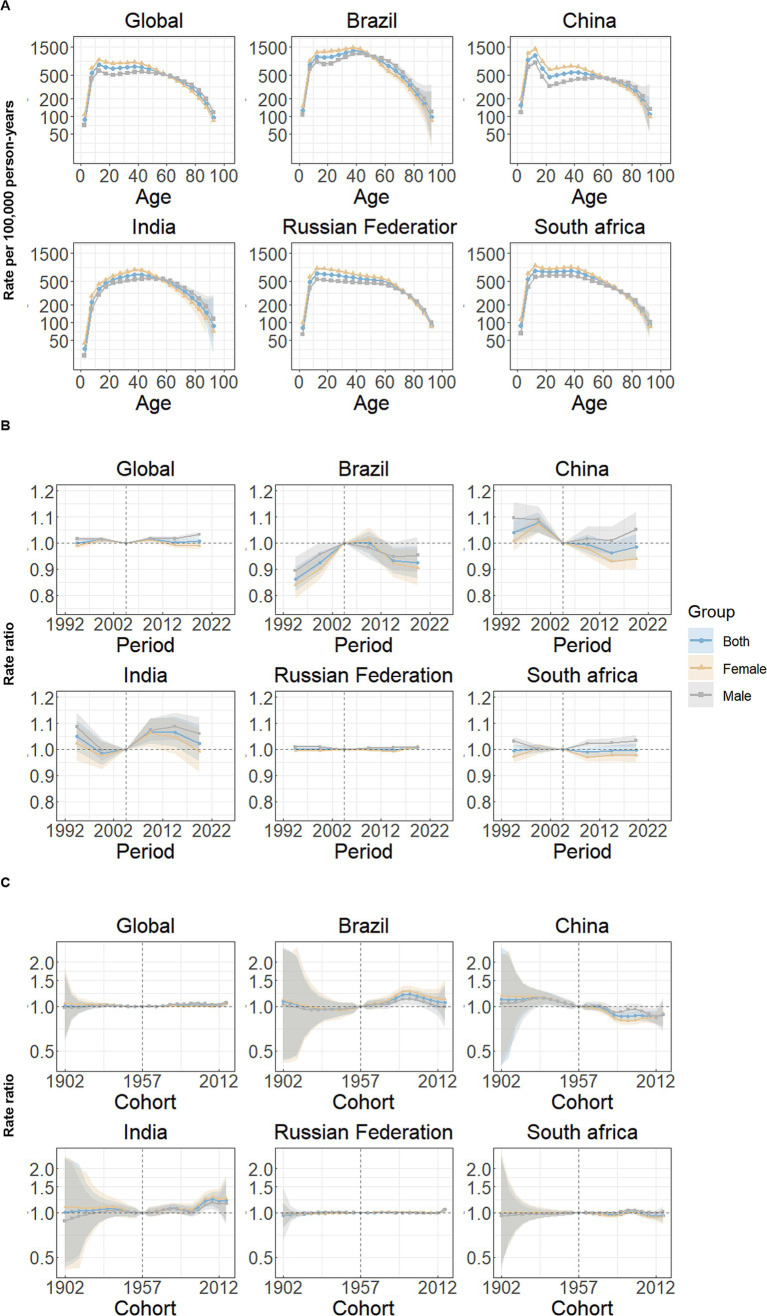
Age, period and cohort effects on anxiety disorders incidence in global and BRICS. **(A)** Age effects are shown by the fitted longitudinal age curves of incidence rate (per 100,000 person-years) adjusted for period deviations. **(B)** Period effects are shown by the relative risk of incidence rate (incidence rate ratio) and computed as the ratio of age-specific rates from 1992–1996 to 2017–2021, with the referent period set at 2002–2006. **(C)** Cohort effects are shown by the relative risk of incidence rate and computed as the ratio of age-specific rates from the 1902 cohort to the 2017 cohort, with the referent cohort set at 1957. The dots and shaded areas denote incidence rates or rate ratios and their corresponding 95% CIs.

Figure 2 illustrates the estimates of Age-Period-Cohort (APC) effects derived from the APC model for global and BRICS countries. Overall, a similar age effect pattern was observed across all nations, and the incidence of anxiety disorders showed two upward and downward trends with age in the reference cohort after adjusting for period effects, similar to an “M-shaped” curve. Among both male and female individuals, individuals aged 10–14 years had the highest incidence rate of anxiety disorders, and the second peak occurred at ages 35–39 years (Figure 2A).

Figure 2B shows the estimated period effects by sex during the whole study period. Globally, period effects have remained relatively consistent over the past three decades, and similar frameworks can be shown in Russian Federation. In Brazil, period effects exhibit a continuous decline, suggesting effective control of anxiety disorders incidence rates over time; while India demonstrates an upward trend, with the risk increasing and remaining above 1. Particularly, the period effects showed a downward trend for female individuals and an upward trend for male individuals from 2007–2011 to 2017–2021 in China and South Africa, compared with reference period (2002–2006).

Cohort effects have remained relatively stable both globally, and in Russian Federation and South Africa. Brazil and India demonstrate varying increasing trends in the successive birth cohort over the past 30 years, especially after reference cohort (1955–1959). However, the cohort RR showed decreasing patterns for both sexes at the observed birth cohorts, overall (Figure 2C).

## Discussion

This study, employing the GBD 2021 database, uncovers a marked escalation in the incidence of anxiety disorders globally and within the BRICS countries from 1992 to 2021. The analysis reveals a 73.44% surge in case numbers and a 21.17% uptick in the age-standardized incidence rate, with notable variations among BRICS countries. Additionally, the current findings underscore the multifaceted nature of anxiety disorders epidemiology, and reflect substantial health disparities and potential priority-setting of depressive disorders incidence in the three dimensions of age, period and birth cohort in BRICS countries.

The observed increase in anxiety disorders incidence over the three decades, as evidenced by our findings, aligns with previous studies that have documented rising mental health issues globally (2, 17). In particular, India’s dramatic rise by 113.30% underscores the urgent need for targeted interventions in rapidly urbanizing countries where lifestyle changes and socio-economic pressures are mounting (18). Comparatively, Brazil’s significant increase of 41.15% could be attributed to its complex socio-economic transformations and public health policies, which have both positively and negatively impacted mental health outcomes (19, 20). China’s modest increase of 2.79% might reflect its unique cultural attitudes towards mental health and the effectiveness of its healthcare reforms in mitigating the rise of mental disorders (21). The differential trends in anxiety disorders incidence across age groups suggest that interventions must be age-specific. The predominantly positive local drift values in younger and older age groups globally, and particularly in Brazil and India, indicate a critical need for early mental health interventions and geriatric care (18, 22). Conversely, the negative drift in China and South Africa for most age groups could either reflect successful interventions or underreporting, necessitating further investigation (21, 23). The gender disparity observed, with more age groups showing positive net drift values for males, highlights the importance of gender-sensitive mental health policies.

Anxiety disorders exhibit significant geographical variation in incidence rates, offering policymakers a unique opportunity to evaluate their countries’ specific characteristics and use this information to guide decisions, considering their relative standings. Despite extensive research, the triggers for anxiety disorders, particularly the influences of age, period, and cohort effects, remain incompletely understood. Addressing these complexities, our study focuses on analyzing patterns of anxiety disorder incidence globally and specifically within BRICS countries, utilizing the APC model. The observed dual peaks in anxiety disorder incidence at ages 10–14 and 35–39 suggest that these transitional periods—adolescence and mid-life—are times of increased stress in BRICS societies. Adolescents aged 10–14 show heightened susceptibility to anxiety disorders, influenced by their developing temperament, family dynamics—including parental education and separation—childhood adversity, and negative life events (24, 25). Concurrently, individuals aged 35–39 enter a critical life phase characterized by intensified stress, including economic and occupational pressures, leading to increased susceptibility to anxiety disorders. Research well documents the association between prolonged economic stress and increased symptoms of worry, anxiety, and panic (26). Our investigation reveals a significant gender discrepancy in the incidence of anxiety disorders, with females showing a higher propensity for these conditions. This discrepancy may be attributed to sex-specific hormonal influences. Empirical evidence indicates that females are more prone to anxiety disorders during various stages of their reproductive lives, including puberty, menstruation, pregnancy, postpartum, and menopause (27). These periods are characterized by significant hormonal shifts, suggesting a role for sex hormones in the initiation, progression, and persistence of anxiety disorders among females. Additionally, females’ heightened sensitivity to stress and traumatic experiences may further contribute to the observed gender disparity (28).

The observed continuous decline in period effects for anxiety disorders in Brazil indicates a potentially effective public health policy and mental health infrastructure that has evolved significantly over recent decades. Such success could be attributed to the implementation of the Psychiatric Reform that started in the late 1980s, aiming to shift care from hospital-centric models to community-based settings, which may have contributed to improved accessibility and early management of mental health issues (25, 29). Additionally, Brazil’s focus on integrating mental health into primary care under the Family Health Strategy might also play a crucial role in this positive trend (30). Despite these advancements, the observed increasing cohort effects signal that newer generations are still experiencing rising incidences of anxiety, possibly driven by societal pressures such as economic instability and urban stressors (31), suggesting a need for continued adaptation and strengthening of mental health services to address these emerging challenges. The stable period and cohort effects in the Russian Federation might suggest an overall stagnation in the evolution of mental health policies, in contrast to other BRICS nations. The lingering anxiety levels might have been compounded by the economic and social instability following the Soviet era, with mental health being overshadowed by other health and economic priorities (32). The consistent exposure across cohorts could also imply a generational transmission of psychological stress, potentially exacerbated by chronic economic strains and political fluctuations (33). This calls for a reevaluation of current mental health strategies, focusing on modernizing and expanding mental health services to reduce these persistent anxiety levels across all age groups. India’s upward trend in period effects suggests an increasing burden of anxiety disorders, likely paralleled by the rapid socio-economic changes and urbanization that have characterized recent decades in the country (34). This increase might also reflect the growing awareness and decreasing stigma associated with mental health issues, leading to more people seeking help. However, the health infrastructure may still be under-equipped to handle this growing burden (35), emphasizing the need for robust mental health policies and the integration of mental health care into primary health care systems. The rising cohort effects indicate that younger populations are particularly affected, which could be due to pressures from educational and employment challenges in a highly competitive environment (25, 36).

The differing period effects between genders in China highlight the impact of cultural, social, and possibly occupational factors that differentially affect males and females. Economic transformations and the shift towards high-pressure educational and employment environments may contribute to these trends (37). While the government has made strides in mental health awareness and treatment, the specific needs of different genders may not be adequately addressed, pointing to a need for gender-sensitive mental health interventions. The stable cohort effects suggest that despite rapid societal changes, the prevalence of anxiety disorders has not significantly shifted across generations, possibly indicating effective community and family support systems that mitigate these disorders (38, 39). Similar to China, the variance in period effects between genders in South Africa could reflect the differing roles and stressors faced by males and females, influenced by ongoing economic disparities and social challenges (25). The persistent socio-economic inequality and historical trauma from the apartheid era continue to impact mental health across the population (40, 41). The stability in cohort effects indicates a continuous exposure to these stressors across generations, underscoring the need for interventions that address both current and historical determinants of mental health. Public health strategies focusing on reducing inequality and improving social determinants of health are essential to combat these entrenched issues.

In contrast to the findings of the GBD 2019 reports (2), our study offers a nuanced analysis of disease trajectories by applying age, period, and cohort effects. This approach differentiates among various global incidence risk factors, with particular emphasis on BRICS nations. A significant enhancement in our research is the detailed quantification of shifts in the incidence age distributions and the initial age of onset from 1992 to 2021, encompassing both global and BRICS-specific data. Employing this methodology, we clarify temporal trends in incidence within particular age groups while accounting for period- and cohort-specific influences. The detailed APC analysis provides a robust framework for understanding the temporal dynamics of anxiety disorders, guiding future policy-making and clinical interventions. Nonetheless, our study acknowledges several limitations. First, data compilation for the GBD 2021 draws from diverse sources such as surveys, registries, and administrative records, which differ in quality and completeness, potentially introducing bias and uncertainty into our conclusions. Second, the GBD database often relies on modeled estimates for many regions with scarce direct data. These models are based on assumptions that might not hold universally, especially in contexts like anxiety disorders, where disease burdens are influenced by varying cultural, genetic, and environmental factors. Third, the use of age–period–cohort analysis could lead to ecological fallacies. Therefore, we have formulated several scientifically plausible hypotheses regarding the causal relationships underlying the temporal patterns of anxiety disorders incidence, supported by available information and existing evidence.

## Conclusion

In conclusion, our study underscores the complex interplay of age, period, and cohort effects on anxiety disorders within the BRICS nations. Each country exhibits unique trends that reflect their specific socio-economic, cultural, and historical contexts, suggesting that tailored public health strategies are essential for effectively addressing and managing the incidence of anxiety disorders in these diverse settings. Future research should delve deeper into the impact of specific policies and interventions, ensuring a comprehensive approach to managing anxiety disorders in these rapidly evolving nations.

## Data Availability

The datasets presented in this study can be found in online repositories. The names of the repository/repositories and accession number(s) can be found below: http://ghdx.healthdata.org/gbd-results-tool.

## Footnotes

^1^ https://ghdx.healthdata.org/gbd-2021
